# The effects of shoe type on lower limb venous status during gait or exercise: A systematic review

**DOI:** 10.1371/journal.pone.0239787

**Published:** 2020-11-25

**Authors:** Lucie Lerebourg, Maxime L'Hermette, Charlotte Menez, Jeremy Coquart

**Affiliations:** 1 Normandie Univ, UNIROUEN, CETAPS, Rouen, France; 2 Orthodynamica Center, Mathilde Hospital 2, Rouen, France; University of Illinois at Urbana-Champaign, UNITED STATES

## Abstract

This systematic review evaluated the literature pertaining to the effect of shoes on lower limb venous status in asymptomatic populations during gait or exercise. The review was conducted in accordance with PRISMA (Preferred Reporting Items for Systematic Reviews and Meta-Analyses) guidelines. The PubMed-NCBI, EBSCO Host, Cochrane Library and Science Direct databases were searched (March 2019) for words around two concepts: shoes and venous parameters. The inclusion criteria were as follows: (1) the manuscript had to be published in an English-language peer-reviewed journal and the study had to be observational or experimental and (2) the study had to suggest the analysis of many types of shoes or orthotics on venous parameters before, during and/or after exercise. Out of 366 articles, 60 duplications were identified, 306 articles were analyzed, and 13 articles met the eligibility criteria after screening and were included. This review including approximately 211 participants. The methodological rigor of these studies was evaluated with the modified Downs and Black quality index. Nine studies investigated the effect of shoes on blood flow parameters, two on venous pressure and two on lower limb circumferences with exercise. Evidence was found that unstable shoes or shoes with similar technology, sandals, athletic or soft shoes, and customized foot orthotics elicited more improvement in venous variables than high-heeled shoes, firm shoes, ankle joint immobilization and barefoot condition. These venous changes are probably related to the efficiency of muscle pumps in the lower limbs, which in turn seem to be dependent on shoe features associated with changes in the kinetics, kinematics and muscle activity variables in lower limbs during gait and exercise.

## Introduction

Although the hemodynamics of the arterial system, which is dominated by the pumping heart, is relatively simple, the hemodynamics of the venous system in the lower limbs, which is dominated by contracting calf muscles, is far more complicated [1–4]. Indeed, many conditions can modify the hemodynamics of the venous system, including foot dysmorphism (*e*.*g*., flatfoot or *pes cavus*), ambient temperature, and even posture (*e*.*g*., standing or seated conditions) [5–12]. In standing and seated positions, gravity increases the hydrostatic pressure [2], causing an accumulation of blood in the lower limbs [4,5]. Several mechanisms return the venous blood accumulated in the lower limbs to the heart, including the activation of the diaphragm pump, the plantar venous pump, and the muscle pumps of the calf and thigh [2,13]. Venous return in the foot is ensured by the plantar venous pump, which propels a quantity of venous blood at each step (in push phase) to both the deep and superficial venous system [14–18]. Therefore, the foot pump should be considered as a real impulse-aspiration system, providing the first push of venous blood in the lower limbs during walking [19,20]. One of the strategies to improve venous function might thus be to act directly on the foot with the use of a device (*e*.*g*., micro-mobile foot compression or a pneumatic compression device) or by wearing certain types of shoe (*e*.*g*., foot orthotics, sandals, heelless shoes…).

For example, Dohm et al. [21] showed that a micro-mobile foot compression device resulted in venous return flow velocity in the posterior tibial and popliteal veins that was greater than the resting velocity. Saggini et al. [9] showed an improvement in the venous emptying capacity of the foot-calf veno-muscular system (in *cavus* foot) with shoes equipped with a specific type of foot orthotic (*i*.*e*., visco-elastic insoles). More recently, Lopéz-Lopéz et al. [22] demonstrated an increase in venous return in the feet with shoes equipped with orthotics (*i*.*e*., customized with ethylene-vinyl acetate material). However, in addition to these technologies (*i*.*e*., foot compression devices, insoles and orthotics) to improve venous function, there are now shoe products (*e*.*g*., sandals, heelless shoes) that can improve venous flow [23–25] and might optimize recovery [26]. Indeed, athletes are now wearing "recovery" shoes (*e*.*g*., flip-flops, sandals) between series of exercise and after training session or competition.

Currently, shoes come in a wide range of types (*e*.*g*., open-toed sandals, flat shoes, heeled shoes, safety shoes, athletic shoes…) and each type uses a different technology (*e*.*g*., rocker soles, unstable footwear, foot-bed, balance pods and multi-density soles) to reduce discomfort or pain [27,28] or improve recovery [26]. Moreover, several reviews have indicated that shoes particularly affect gait kinematics and kinetics both acutely and chronically [29–34]. For example, some unstable shoes have been reported to improve upright walking posture, reduce lower limb joint motion and loading, and/or even modify muscle activation patterns [24,33,35,36]. Furthermore, some authors have observed or suggested that unstable shoes have an effect on blood flow or the venous system [24,25,37,38]. However, the quantitative evidence on this last observation is limited and the positive effects of shoes on improving venous return remain unknown.

To date, no study has systematically reviewed the effects of a range of shoe types (including foot orthotics, insoles, cast shoes…) on lower limb venous status before, during or after exercise. Therefore, the aim of this systematic review was to identify, appraise and summarize the available evidence on the effects of many types of shoes on lower limb venous status in asymptomatic populations.

## Materials and methods

This systematic review was executed in accordance with PRISMA (Preferred Reporting Items for Systematic Reviews and Meta-Analyses) guidelines (S1 Checklist and S1 Fig) [39]. Moreover, the review protocol was registered in the International Prospective Register of Systematic Reviews (PROSPERO registration number: CRD42019121613).

### Identification

A systematic search of the research literature was conducted to look for studies that assessed the effects of all types of shoes on lower limb venous status during exercise. No restriction in terms of period have been retained. Therefore, this search was undertaken in the PubMed-NCBI (1957–2019), EBSCO Host (1981–2019), Cochrane Library (1978–2019) and ScienceDirect (1973–2019) databases. The search was conducted independently by three of the authors (LL, CM, JC) in March 2019 from keywords identified by all co-authors.

**Fig 1:**
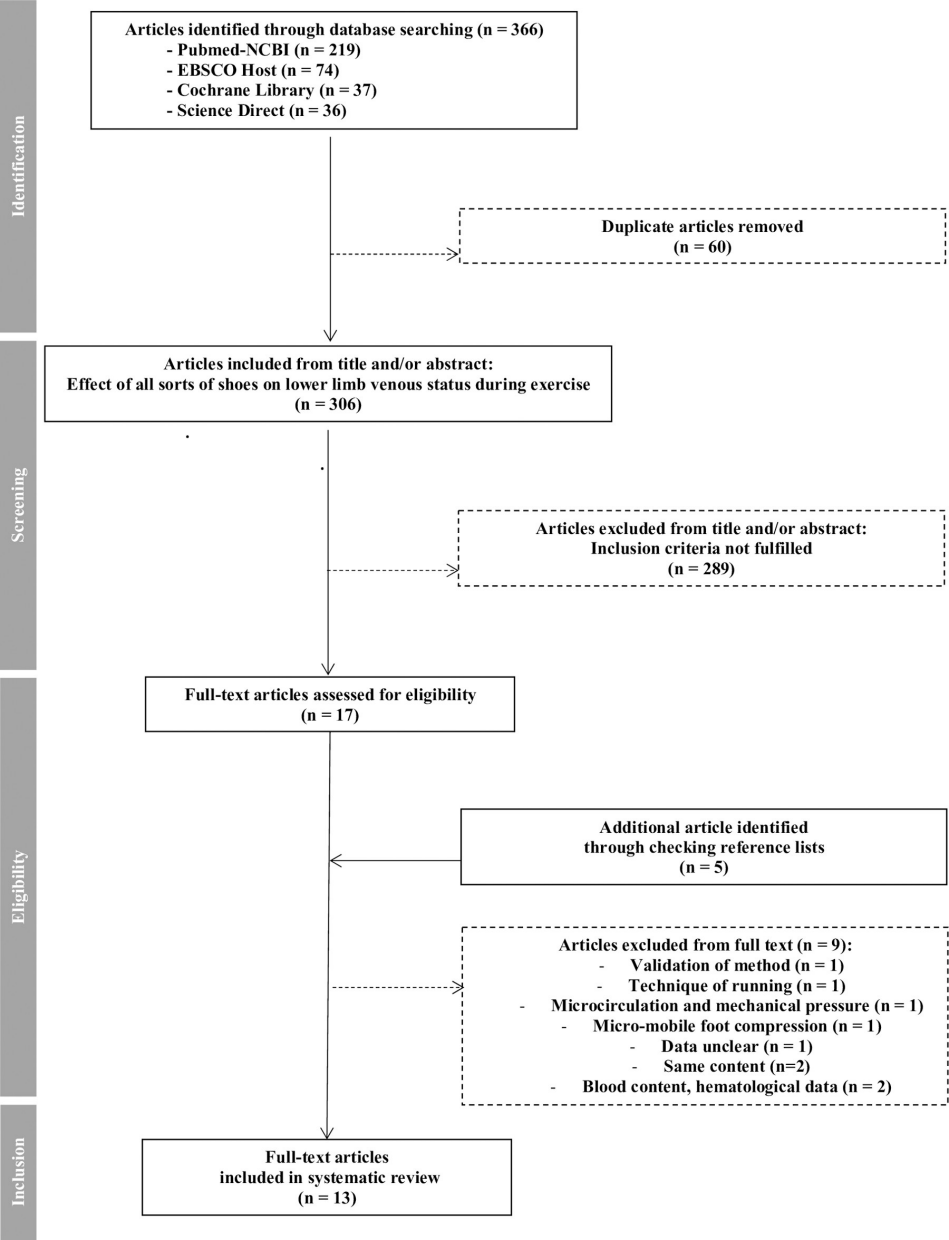
Flow diagram of study selection. Figure presents the flow of information through the different phases (identification, screening, eligibility and inclusion) of a systematic review.

These keywords were based on two concepts: shoes and venous parameters (Table 1). All MeSH (Medical Subject Headings) terms associated with each concept (*i*.*e*., synonyms) in the National Library of Medicine’s (NLM) controlled vocabulary thesaurus were collected. Moreover, the authors completed this list of terms/synonyms with keywords that are frequently used in the literature to represent both concepts. Then, all identified terms/synonyms in MeSH and the keywords were combined with Boolean operators (“AND” and "OR"), and the search was conducted in the PubMed-NCBI, EBSCO Host and Cochrane Library databases (Table 1). However, for the ScienceDirect database, only the MeSH heading (not the terms/synonyms) and keywords were used because this database is unable to support more than eight Boolean operators per field (Table 1). The search string used the Boolean operator “AND” to combine the two concepts and “OR” to provide a comprehensive set of terms for each concept (Table 1). The search was conducted for titles, abstracts and keywords of citations in each database. This revealed 366 manuscripts. Once duplicate citations were removed (n = 60), 306 articles were analyzed (Fig 1).

**Table 1:** Search strategy.

Databases	Concept 1	Concept 2
PubMed-NCBI, EBSCO Host and Cochrane Library	(*footwear* OR **shoe** OR **orthoses, foot** OR **foot orthosis** OR **orthosis, foot OR foot orthotic devices** OR **device, foot orthotic** OR **devices, foot orthotic** OR **foot orthotic device** OR **orthotic device, foot** OR **orthotic devices, foot** OR **foot arch supports** OR **arch support, foot** OR **arch supports, foot** OR **foot arch support** OR **support, foot arch** OR **supports, foot arch** OR **orthotic shoe inserts** OR **insert, orthotic shoe** OR **inserts, orthotic shoe** OR **orthotic shoe insert** OR **shoe insert, orthotic** OR **shoe inserts, orthotic** OR **orthotic insoles** OR **insole, orthotic** OR **insoles, orthotic** OR **orthotic insole** OR *orthopedic shoe* OR *therapeutic shoe*)	(*venous* OR *blood flow* OR **vein)**
Science Direct	(*footwear* OR shoe OR foot orthoses OR *orthopedic shoe* OR *therapeutic shoe*)	(*venous* OR *blood flow* OR vein)

Legend. Highlighted words: MeSH (Medical Subject Headings) terms; **words in bold: terms/synonymous associated to MeSH terms for “shoe” and “foot orthoses”**; *words in italics*: *keywords identified by authors and frequently used in the literature*.

### Screening

Following the initial selection of studies, two experts in the field (LL and CM) assessed the eligibility of each manuscript independently in a blinded standardized manner by screening the titles and abstracts. Disagreements were discussed between three authors (CM, LL and JC) and resolved by consensus. To be considered eligible, the manuscript had to be published in a peer-reviewed journal and the study had to be observational or experimental (*e*.*g*., observational, randomized controlled trials …). Letters to the editor, short communications, meeting abstracts, editorials, theses, case reports, reviews and meta-analyses were thus excluded, as were animal studies and studies not published in English. The study also had to suggest the analysis of the effects of several types of shoes or orthotics on venous parameters before, during and/or after physical exercise (*i*.*e*., inclusion criteria). All outcomes relating to shoe fit or characteristics (*e*.*g*., footwear, foot orthotics, insoles, high-heeled shoes …) and hemodynamic venous parameters (*e*.*g*., blood or venous flow, venous pressure, venous reflux, blood rush …) were authorized. Studies were, however, excluded if there were no clear performance measures or if the participants were undergoing medical treatment (excluding orthopedic treatments) or had rheumatic diseases, lacked partial independence in daily activities, or had severe osteoarticular or neurological disease that would compromise posture and walking (*i*.*e*., exclusion criteria).

Following the review of the titles and abstracts, 289 articles were further removed from the analysis (Fig 1).

### Eligibility

Seventeen full-text articles were initially eligible. The reference lists from these manuscripts were searched manually to identify other possibly eligible articles (CM and LL), and five additional manuscripts were identified. These manuscripts were then screened, according to the procedure described above. None of these new articles was excluded on the basis of the titles and/or abstracts. Therefore, 22 full-text articles were evaluated. Based on the information in these articles, two authors (LL and CM) used a standardized form to select the manuscripts eligible for inclusion in the systematic review. This form was used to determine whether the information provided in the title and abstract matched the text content and met the inclusion criteria (*i*.*e*., shoes or insoles, analysis of venous hemodynamic parameters before, during and/or after exercise …). Ultimately, the review included 13 studies with approximately 211 participants.

### Inclusion

From the 13 included articles, the authors (LL and JC) extracted the following information: study identity (*i*.*e*., first author and year of publication), participants (*e*.*g*., number, status, sex, age), study design (*e*.*g*., randomized, non-randomized), type of shoe or insole intervention (*e*.*g*., protective, athletic, high-heeled, custom orthotics …), type of exercise or protocol (*e*.*g*., walking, prolonged standing position, treadmill running …), duration of adaptation (*e*.*g*., several minutes, days…), venous hemodynamic variables analyzed (*e*.*g*., blood flow, venous pressure, circumferences), methods or materials (*e*.*g*., plethysmography, Gulick tape measure, Doppler ultrasound), and data to assess venous activity.

### Risk of bias and quality appraisal

The methodological rigor of the studies was evaluated with the modified Downs and Black quality index (QI) [40]. The QI is a 27-point checklist [40–42] to assess the methodological quality of both randomized controlled trials and non-randomized studies [42]. This tool has the following four subscales: reporting (items 1–10), external validity (items 11–13), internal validity (items 14–26), and power (item 27). The wording of item 13 was modified from “were the staff, places, and facilities where the patients were treated, representative of the treatment of majority of patients receive?” to “was the intervention representative of those that are used in clinical practice/commercially available?” [33]. The maximum score was 28 because item 5 had a possible score of 2 if scored “yes”, 1 if scored “partially” and 0 if scored “no” or “unable to determine.” For all other items, “yes” was scored 1 and “no” and “unable to determine” were scored 0. Based on the risk of bias assessment scores, studies were classified as very high quality/very low risk of bias (26–28), good quality/low risk of bias (20–25), fair quality/fair risk of bias (15–19) or poor quality/high risk of bias (≤ 14) [43]. Higher scores indicated less risk of bias than lower scores. The QI has been shown to have high internal consistency (Kuder-Richardson Formula 20 = 0.89), test-retest reliability (r = 0.88), and inter-rater reliability (r = 0.75) [40].

Two authors (LL and CM) independently appraised all studies in each of the domains of this tool. Disagreements during the quality assessments were discussed until consensus was reached. If consensus was not reached, a third reviewer (JC) provided the final judgment.

## Results

The search strategy revealed that 13 articles met the inclusion criteria (Fig 1).

Two studies compared unstable shoes with regular shoes (*i*.*e*., conventional, flat-bottomed shoes) or barefoot condition [24,37]. Four studies compared athletic shoes (*e*.*g*., soft shoes) with firm shoes (*i*.*e*., clogs), heelless shoes or barefoot condition [25,44–46]. Two studies compared high-heeled shoes (*i*.*e*., stilettos) with different types of high heel (*i*.*e*., medium heels, platform high heels) or barefoot [47,48]. Two studies analyzed the influence of protective shoes (*e*.*g*., all-rubber firefighting shoes) during exercise [49,50]. One study compared sandals with conventional shoes and barefoot [23]. Another study analyzed the effect of ankle joint immobilization with different shoes (*i*.*e*., cast, pneumatic walking boot) or positions of immobilization (*i*.*e*., neutral, equinus) [51]. Only one study collected data on insoles (*i*.*e*., customized foot orthotics) *vs* without insoles [22]. Five studies used consistent treadmill exercise (walking or running) [23,25,47,49,50], one used running exercise [46], five used an upright standing exercise (*e*.*g*., simulated work) [22,24,37,44,45], two used tip-toe movements [47,48] and one asked participants to simulate gait [51].

The participant characteristics, study design, interventions (shoe conditions), gait type (type of exercise), duration of adaptation and venous variables analyzed in each study are presented in Table 2. The main statistically significant changes from wearing shoes or insoles in the studies are also presented in Table 2. The intervention duration, whether with shoes or insoles, varied between several minutes to 8 weeks. The participants were between 20 to 50 years old, and 54.3% were female (n = 119) and 45.7% were male (n = 100). All 13 studies measured hemodynamic venous data and at least one of the venous variables: blood flow, venous pressure or circumference (Table 2). As Table shows, the results of eight studies indicated that intervention with certain shoes resulted in positive blood flow effects (*i*.*e*., increase in venous flow or decrease in edema formation) [22,24,25,44,46], positive pressure effects [23,47], or positive circumference effects (*i*.*e*., decrease in circumferences) [37]. One study emphasized the harmful effects (increase in residual volume in legs) of some shoes (high-heeled shoes) on venous flow [48]. Three other studies reported that the blood flow changes [49–51] appeared to be dependent on both the shoe type and the exercise conditions (*e*.*g*., humidity, temperature, immobilization, weight-bearing). Finally, the studies of Karimi et al. [37] and Lin et al. [45] reported that some shoe types did not have significant clinical effects on venous data, such as circumferences (Table 2).

**Table 2:** Characteristics and primary outcomes of studies.

Author (year)	Participant characteristics	Study design	Intervention/Shoe conditions	Type of Exercise/Gait type	Duration of adaptation	Venous variables analyzed/ Outcome measure(s)	Significant results/Main findings
Knight and Lewis (1977) [23]	9W and 3M Age (range): 29 (20–38) years Individual sample data: • Those who had never worn sandals • Those who habitually wore sandals	Randomized sequences	Barefoot/ Normal shoes/ Sandals	Treadmill walk	14 days	Venous pressures (mmHg) by pressure transducers	Initial: No significant difference in the exercise venous pressure in non-wearers and a significantly lower venous pressure in accustomed wearers when walking with sandals (pressure similar to that of barefoot walking). After 14 days: A significantly lower venous pressure while walking in sandals compared to shoes in both groups. No significant difference in the exercise pressure when walking barefoot compared to that in sandals.
Hansen et al. (1998) [44]	8 W Age (range): 24 (21–29) years Whole sample data	A balanced block design	Clogs (without heel support as the hard shoe)/Sport shoes (soft shoes)	Simulated work (standing and standing/walking)	2 hours/work	Blood flow by hydro- plethysmography (FV and VV)	Standing work: Significant reduction in edema formation in soft shoes condition (from 3.2% to 2.8% on average). Standing/walking work: Significant decrease in the edema formation (from 2.2 to 1.2%) for soft shoes condition.
Yamamoto et al. (2000) [25]	6 M Age (SD): 19.5 (± 4.0) years Whole sample data	Randomized	Regular shoes (regular athletic shoes)/ Heelless shoes	Treadmill walk (at a speed of 60, 80, 100 and 129 m.min^-1^)	10 min/test	Blood flow (CBF) by venous occlusion plethysmography	Blood flow: A significantly higher CBF at the speed of 80 m.min^-1^ for heelless shoes than regular shoes. No significant difference in CBF in regular shoes or heelless shoes at walking speeds of 60, 100 and 120 m.min^-1^.
Potério-Filho et al. (2006) [47]	10 W Age: 20–50 years Individual sample data: • Genu Valgum and flat feet (n = 5) • No orthopedic problems (n = 5)	N/A	Barefoot/ High-heeled shoes (stiletto)	Tip-toe movements Treadmill walk	1 or 10 extension/flexions 1 min/ treadmill walk test	Venous pressures (mmHg) by APG (variations of the pressure, hydrostatic leg vein pressures)	No statistical difference between the variation in the cuff pressure values when persons performed 1 or 10 tip-toe foot movements. Statistical differences for the variation of the cuff pressure during static movements (41.05 mmHg), walking barefoot (26.65 mmHg) and walking with high-heeled shoes (52.2 mmHg). Significantly higher variations in the cuff pressure readings were obtained at the leg when compared with walking barefooted (during walking with high-heeled shoes). Significant difference for the final hydrostatic pressure at the end of walking between the 2 groups. Significant difference in the final hydrostatic pressure, for all individuals, when walking with high-heeled shoes (53.5 mmHg) and barefoot (61.5 mmHg).
Irzmańska et al. (2011) [50]	10 M (firemen) Age: 20–45 years Whole sample data	N/A	Protective footwear	Treadmill walk	Several minutes	Blood flow by APG (IR, CT)	Statistically significant changes for the APG parameters and temperature-humidity. Correlation between the level of humidity and temperature and blood flow parameters in the legs (IR, CT) during exercise.
Tedeschi Filho et al. (2012) [48]	30 W Age (range): 26,4 (20–35) years Whole sample data	N/A	Barefoot/ Medium heels (3.5cm)/ Stiletto high heels (7cm)/Platform high heels (7cm)	Tip-toe movements	10 orthostatic flexion and extension/test	Blood flow by APG (EF, RVF, VFI)	EF: Higher values for the barefoot group compared with the other 3 groups. A significant decrease in both of the 7-cm high-heeled groups compared with the barefoot. RVF: Significant increase in the groups wearing high heels (stiletto and platform) and medium heels (3.5 cm) compared with barefoot group. Despite the lack of statistical significance, the medium-heel group showed lower values compared with the 2 high-heeled groups. No difference between the 2 7.0-cm high-heeled groups for this parameter. VFI: Similar values in the 3 situations evaluated.
Sousa et al. (2012) [24]	30 W Age: 20–50 years. Individual sample data: • Experimental group with unstable shoe (n = 14), 34.6 (± 7.7) years • Control group with conventional shoe, (n = 16), 34.9 (± 8.0) years	Randomized trials	Barefoot/USW/CSW	Upright standing	8 weeks (Experimental group only: wear USW 8h/day for 8weeks) 30s/ data acquisition of trial	Blood flow by duplex ultrasonography (CFV, PV)	No significant difference in the mean flow rate at CVF and PV between the experimental and control groups for both the first and second evaluations. A comparison between measurements taken with and without the unstable shoe shows a higher level of venous return with the unstable shoe in both groups during the first and second evaluation in PV and CFV. Venous return increased in subjects wearing USW before and after 8 weeks. No differences between the first and second evaluation, both with and without the unstable shoe, as for the influence of 8 weeks of unstable shoe wearing. The same was verified in subjects who wore conventional footwear.
Lin et al. (2012) [45]	10 M Age (SD): 30.2 (± 2.9) years Whole sample data	Randomized	Barefoot/ Sport shoes	Upright standing	4 hours (1 hour/experimental session)	Circumferences (thigh and shank) by Gulick Tape	No significant difference in thigh and shank circumferences between the 2 shoe conditions, but the difference in shank circumference was highest when a subject stood for a prolonged period on the hard floor while wearing sport shoes.
Irzmańska et al. (2014) [49]	30 M (firefighters), Age (range): 30.7 (25–35) years Work experience (5–10 years) Whole sample data	N/A	All-rubber firefighter footwear (with protective elements)	Treadmill walk	1 hour 45 minutes	Blood flow by APG (IR, SR, CW, CT, ABF)	Statistically significant parameters of blood flow in the lower limbs were the IR SR, CW and ABF during the 3 phases (warm-up, exercise and rest) with a rise in values of the IR, SR, CW and ABF during exercise as compared to the initial rest phase and a decrease in the relaxation phase. No significant difference for the CT, remained at similar levels.
Craik et al. (2015) [51]	6 M and 4 W Age (range): 38 (27–61) years Whole sample data	N/A	Without ankle joint immobilization/ ankle immobilized in a neutral cast/ankle immobilized in a pneumatic walking boot (in both neutral and equinus immobilization)	Simulation of the gait cycle	30 repetitions/minute	Blood flow (VBF velocity) by duplex ultrasonography (Time averaged peak velocity, time averaged mean velocity, total volume flow)	No significant reduction in VBF measurements between full weight-bearing without ankle joint immobilization and full weight-bearing in a neutral cast or neutral pneumatic walking boot. Reduction of VBF when partial weight-bearing (50%) and when full weight-bearing in a pneumatic walking boot in *equinus*.
Wezenbeek et al. (2017) [46]	25 (experienced runners), 15 M and 10 W, Age (SD): 34.5 (± 10.2) years Whole sample data	Non-randomized order (running activity)	Barefoot/ Shod (Pearl Izumi shoes, offset of 7 mm)	Run	30 min (10 min/phase: rest, barefoot run, shod run)	Blood flow by oxygen-to-see device	A significant increase in blood flow after barefoot and shod running compared to initial tendon blood flow (42.6% after barefoot running and 61.7% after shod running).
Karimi et al. (2017) [37]	10 M Age (SD): 25.3 (± 1.49 years) Whole sample data	Randomized	Barefoot/Flat-bottomed shoe (stable)/USW	Upright standing	2 hours/shoe condition (session). One-week interval between each test session	Circumferences (lower legs) by Gulick tape	An increase in the mean scores of lower leg volume, from pretest to posttest following 2 hr of standing for both legs, under all 3 footwear conditions. Results revealed that footwear conditions influenced this value for right and left legs after 2 hr of standing. Results showed that percentage of volume change was reduced by USW in relation to barefoot condition for right and left legs. No significant difference for percentage of volume change between USW and flat-bottomed shoes, or between barefoot condition and flat-bottomed shoes in both legs.
López-López et al. (2018) [22]	10M and 10 W Age (SD): 20 (± 1.62) years Whole sample data	Non-randomized	CFO/ Without CFO	Standing in a relaxed posture	20 days 20 min /session	Blood flow by APG (VFT and EF.	A significant difference between VFT and the EF with CFO utilization versus without use of CFO.

Subjects: number of subjects per sex (W, women; M, men), characteristics; mean age.

Shoe characteristics: CFO, customized foot orthoses; CSW, conventional shoe wearing; USW, unstable shoe wearing.

APG, Plethysmographic.

Venous parameters (ABF, alternative blood flow; CBF, calf blood flow; CFV, venous velocity of the common femoral; CT, crest time; CW, crest width; EF, ejection fraction; Hct, hematocrit; IR, impedance ratio; MCH, mean cellular hemoglobin; MCV, mean cellular volume; FV, foot volume; PV, popliteal veins; RBC, red blood cell; RVF, residual volume fraction; SR, slope ratio; TIBC, total iron-binding capacity; VBF, venous blood flow; VF, venous flow; VFT, venous filling time; VV, vascular volume; WBC, white blood cell).

A summary of the QI scores [40] for each paper is presented in Table 3. The total score of the QI ranged from 16/28 to 23/28, with a mean score of 19.8/28 and a median score of 20/28 for the 13 articles. Five of the studies were of fair quality [23,48–51] and eight were of good quality [22,24,25,37,44–47]. In five studies, the order of the interventions was randomized [23–25,37,45].

**Table 3:** Methodological quality of the included studies assessed by the quality index [40].

Author (year)	Reporting score (score/11)	External validity (score/3)	Bias (score/7)	Confounding (score/6)	Power (score/1)	Global (score/28)
Knight and Lewis (1977) [23]	8	2	3	3	0	16
Hansen et al. (1998) [44]	11	2	5	2	0	20
Yamamoto et al. (2000) [25]	10	2	5	4	0	21
Potério-Filho et al. (2006) [47]	10	3	4	3	0	20
Irzmańska et al. (2011) [50]	8	2	4	3	0	17
Tedeschi Filho et al. (2012) [48]	10	2	4	3	0	19
Sousa et al. (2012) [24]	11	3	5	3	0	22
Lin et al. (2012) [45]	11	2	4	3	0	20
Irzmańska et al. (2014) [49]	10	2	4	3	0	19
Craik et al. (2015) [51]	10	3	4	2	0	19
Wezenbeek et al. (2017) [46]	11	2	5	2	0	20
Karimi et al. (2017) [37]	11	2	5	4	0	22
López-López et al. (2018) [22]	10	3	5	4	1	23

Legend for the global score: excellent (26–28); good (20–25); fair (15–19); and poor (< 14).

## Discussion

The aim of this systematic review was to investigate the effects of various shoes and insoles on lower limb venous status, including blood flow, venous pressure and circumferences, in asymptomatic populations before, during or after exercise. The findings suggest that certain types of shoes can influence venous parameters. However, the studies were quite heterogeneous concerning the shoe types, the duration or type of intervention activity, and the venous parameters studied.

### Effects of shoes on blood flow parameters

Five of the studies focused on unstable shoes [24], heelless shoes [25], athletic shoes [44,46] or insoles [22] and reported an increase in blood flow and venous return [22,24,25,46] or a decrease in edema formation [44] in the lower limbs. It can be assumed that the respective features (*i*.*e*., instability, rocker bottom, soft, customized orthotics) modify the motion or muscle activity in the lower limbs and thus change features of the venous parameters.

Some of the evidence showed that unstable shoes [24] like heelless shoes [25] were associated with kinetic and kinematic changes in lower limb muscle function, as opposed to conventional shoes [30,33]. Indeed, the concept underlying the function of unstable shoes is that instability induces, for example, an increase in the ankle joint’s range of motion during walking [28,52] and an increase in the activity of the *gastrocnemius* [33,36] and *tibialis anterior* [28,36]. Athletic shoes (*i*.*e*., soft shoes) also modify movement patterns, like step frequency (*e*.*g*., increased step/stride length and decreased cadence *vs* barefoot), joint movements (*e*.*g*., decrease in plantar-flexion *vs* barefoot), and muscle activity in the lower limbs [31,32]. These results suggest that blood flow variations are more associated with muscle pump activation. Moreover, it has been demonstrated that athletic shoes *vs* barefoot [46] can also substantially affect subtalar joint movement. Extreme shoe modifications may affect eversion [53] (*i*.*e*., the association of three movements including abduction, pronation and dorsal flexion) [54], and thus the pronation (an aspect of this foot eversion) [55]. Clement et al. [56] first suggested that foot pronation causes partial constriction of the Achilles tendon’s vascular network (whipping of torsional action), and Karzis et al. [57] later showed an increase in vascular resistance and reduced Achilles tendon blood flow with foot overpronation. However, it seems that pronation is mainly due to the foot movement itself [57]. Indeed, the study of Stacoff et al. [53] reported no significant difference in eversion between wearing shoes and going barefoot. This result highlights the observation that shoes do not reflect the movement of underlying bone but only of the shoe and/or skin movement. Thus, it is not the shoe that increases the vascular resistance but rather the pronation movement itself. Eversion may therefore be influenced by the shoe characteristics and/or the exercise conditions and thereby exert an influence on the venous flow [46]. Furthermore, foot orthotics are an effective intervention to reduce rear-foot eversion [58] or the pronated foot-type [59]. They also affect kinetic (*e*.*g*., reduction in the peak and mean ankle eversion moments) and kinematic (*e*.*g*., reduction in peak rearfoot eversion or tibial internal rotation) variables and muscle activity (*e*.*g*., increase in *tibialis anterior* and *peroneus longus* electromyographical amplitudes: EMG) [32,59,60]. There was also evidence reported of the relationship of foot and ankle movements to venous return in the lower limb, where combined active movement produced the highest flow velocities [15,61,62]. This would explain the influence of orthotics use on the increased venous return or the decreased edema formation.

Regional blood distribution is dependent on the muscle fiber type [63–66]. The kinetics of venous return are dependent on contraction frequency, with variations in relaxation periods between the muscle contractions [16,64,67,68]. Walkeling et al. [69] suggested that muscle fiber-type recruitment patterns can also be altered by the choice of shoes and the shoe’s midsole material, and the study of Rao et al. [70] showed lower amplitudes of the *lateral gastrocnemius* and *medial gastrocnemius* fiber lengths in barefoot conditions compared to shod conditions. Thus, the rhythmic contractions of shank or foot muscles [16,64] coupled possibly with an increased range of joint motion [52] while walking with particular shoes like unstable shoes [24,25], athletic shoes [44,46], and orthotics [22] could enhance venous emptying of the foot compared to conventional shoes (*i*.*e*., without this particular feature) or barefoot condition. It is important to note that depending on the type, intensity, duration and order of the exercise protocol, venous return might be affected [64]. For example, in the experiment of Yamamoto et al. [25], calf blood flow was reduced at higher walking speed (120 m.min^-1^). In the study of Wezenbeek et al. [46], the influence of running on blood flow was higher after shod running (running shoes) than after barefoot running, but the running was performed in non-randomized order.

Two studies that focused on high-heeled shoes [48] and ankle joint immobilization [51] reported increased residual volume and a reduced ejection fraction [48] or decreased blood flow in the lower limbs [51]. It seems that such negative results for venous parameters also depend on motion, gait or muscle activity in the lower limbs caused by the features of the shoes (*e*.*g*., high-heel height, as for platform or stiletto shoes, ankle immobilization, cast).

High-heeled shoes were associated with several changes in gait with, for example, a decrease in stride length [71–74], an increase in plantar flexion [72,73,75] or an increase in antagonist muscle activation (*e*.*g*., *medial gastrocnemius*, *rectus femoris*) [34,75]. Yet it has been suggested that this increase in muscle activity [34,75] is not systematically linked to better venous return, because muscle activity is only related to systole and not diastole during calf muscle pumping with high heels. Indeed, the studies of Gardner and Fox [61] and Corley et al. [15] showed an increase in the efficiency of venous drainage in the lower limbs when the ankle kinematics under calf muscle activity were acting in unison with the foot pump. However, it is important to note that other characteristics of high-heeled shoes (*e*.*g*., platform or stiletto) are important, as Tedeschi Filho et al. [48] showed a higher residual volume for platform shoes compared to stilettos despite the same heel height (7 cm). Thus, the variation in venous return/hemodynamic changes could be linked to kinematic differences [72] like restricted plantar and ankle joint movements with platforms compared to stilettos.

Regarding ankle joint immobilization, Craik et al. [51] showed a decrease in blood flow in the lower limbs under partial weight-bearing conditions (50%) and full weight-bearing in a pneumatic walking boot in equinus. These results can be explained, as previously, by a decrease in the efficiency of muscle pumps (calf and foot). Ankle kinematics work in synergy with the foot pump and each foot compression with weight-bearing permits an effective propulsion of blood [15]. It appears that ankle joint immobilization and a pneumatic walking boot in equinus are not as effective as the natural foot pump in producing venous flow, possibly due to their inability to stress the foot vein plexuses [76], and thus neither provides an active muscle pump [77]. Moreover, when the ankle is immobilized, the movements are clearly limited and flow velocities may thus be decreased. Indeed, Sochart and Hardinge [62] showed that combined active movements produced the highest flow velocities compared to passive movements and are thus required to promote significant increases in venous blood flow.

Last, studies of protective footwear (firefighter footwear with a wool liner) were included [49,50]. In both, the footwear was a model designed for firemen and used in firefighting and emergency actions. The results of these studies showed a correlation between the humidity and temperature and the blood flow parameters in the legs during exercise [49,50]. One of the studies reported an increase in the blood flow in the lower limb vessels due to temperature and humidity inside the footwear and the physical exertion [49]. Indeed, the human foot sweats in response to significant exercise and thermal stimulation [78]. It is well known that the typical vascular reactions in the human body depend on body temperature [64,79], and in response to the thermal effect of protective footwear during prolonged exercise, the body reacts by the dilation of skin vessels and increased skin blood flows. These protective shoes nevertheless can generate excessive heat, which may result in an excessive load on the vascular system and suggests the deterioration of blood flow in the lower limbs.

### Effects of shoes on venous pressure

Two studies that focused on sandals [23] and high-heeled shoes [47] reported a decrease in venous pressure [23,47] and an increase in the variation of venous pressure [47]. The conclusions drawn regarding this variable were similar to those for blood flow. Based on the features (*e*.*g*., soft or hard sole, midsole, density, rubber sole, rocker …) and the shoe type, there is some evidence that the range of motion, joint movement, and the muscle or muscle pump activity are affected [30–32,34,80–84] by sandals [55,83,85,86] *vs* conventional shoes [86–88] and by high-heeled shoes [73,89] *vs* barefoot [34,72,75,90]. Several studies have shown an increase in the activity of *soleus*, *peroneus longus* [84] or *tibialis anterior* during exercise with sandals and flip-flops [83,84,91]. Furthermore, Carl and Barrett [92] demonstrated higher peak plantar pressures with flip-flops than athletic shoes but lower pressures than barefoot. These modifications in muscle activity or plantar pressure could explain the increase in muscle pump activity [16] and consequently the significantly greater fall in venous pressure (*i*.*e*., significant difference) with sandals exercise, approximating barefoot walking, than with shoes. The lack of difference (*i*.*e*., no significant difference) in venous pressure between sandals and barefoot conditions (*i*.*e*., pressure similar) may be explained by broadly similar patterns of foot motion during walking with no significant changes in kinetic or kinematic variables [55,87,88].

We note that one of the major factors that influence muscle activity is walking speed [93]. Indeed, beyond a certain walking speed (*i*.*e*., 100m.min^-1^), Kayaga et al. [94] reported the decrease in calf blood flow when walking. Moreover, gait velocity increases antagonist activation [95]. However, as noted by Blackburn et al. [84], an increase in muscle activity and most likely the level of agonist-antagonist co-contraction may not be positive outcomes. For example, an increase in ankle invertor strength without a concomitant increase in evertor strength could predispose the ankle joint to inversion after invertor-evertor coactivation. This might be the origin of the lower efficiency of muscle venous pumps in the lower limbs.

The results of Potério-Filho et al. [47] (*i*.*e*., improved function of the leg muscle pump, which results in lower leg venous pressure during walking) also seem contrary to the general assumption that walking with high-heeled shoes is detrimental to the efficiency of the leg muscle pump. Tedeschi Filho et al. [48] indicated that high heels have a deleterious action on venous return and thus tend to cause venous hypertension in the lower limbs, whereas Potério-Filho et al. [47] reported lower leg venous pressure and greater variation in pressure during walking in high heels compared to barefoot. It has been established that walking in high-heeled shoes is a common cause of venous complaints such as hypertension, pain, fatigue, or the feeling of heavy legs and may be a causal factor of venous disease symptoms. The proposition of Potério-Filho et al. [47] that an increase in muscle contractions or greater expulsion of blood that generates higher pressure in the muscle compartments during walking in high heels might be questioned. Indeed, although several studies have shown an increase in muscle activity with high heels [34,75,96,97] or joint restriction [29,98,99], this does not automatically indicate better efficiency of the venous pump (linked with systole and diastole during calf muscle pumping) during walking and in principle this does not tend toward a better venous return [3,16].

It is important to recall here that a difference in the protocols for high heels (*i*.*e*., tip-toe movements *vs* walking) in the studies of Potério-Filho et al. [47] and Tedeschi Filho et al. [48] might have affected plantar movements and muscle activity differently and thus affected blood flow parameters or muscle pump efficiency differently [16,95]. It also seems that changes in the muscle pump, as in the blood flow, can modify venous pressure.

### Effects of shoes on circumferences

Two studies evaluated circumferences during upright standing exercise. Karimi et al. [37] tested various footwear conditions (*i*.*e*., barefoot, stable shoe, unstable shoe) and found lower reduction in lower leg circumferences with unstable shoes *vs* barefoot only, and Lin et al. [45] showed no significant difference in leg circumferences between barefoot and athletic shoes. We therefore note that in addition to similar exercise conditions (*i*.*e*., upright standing), these two studies showed similar results, especially between stable shoes or athletic shoes *vs* barefoot condition, with no significant difference [37,45].

Thus, the findings of the least amount of swelling swelling (*i*.*e*., with unstable shoes *vs* barefoot only) in leg circumferences [37] could be explained by the features of the shoes, especially unstable shoes, which eliminate stasis and promote venous return. As noted, the rocker sole design, which decreases the degree of the stability surface more than other shoe conditions, induces modifications in the kinetics, kinematics, and muscle activity of the lower limbs [28,30,33,36,52]. Several studies have shown an increase in the activity of *gastrocnemius* [33,36] and *tibialis anterior* [28,36] and the range of motion [28,52] with unstable shoes. Moreover, the findings of Karimi et al. [37], in agreement with those of Sousa et al. [100], reveal that standing with unstable shoes also leads to significantly lower muscle co-contraction in the lower leg (*e*.*g*., *tibialis anterior* and *gastrocnemius*) compared to barefoot only, while other studies have shown an increase in muscle co-contraction during walking at self-selected speed with unstable shoes compared to regular shoes [101,102]. These kinetic, kinematic and muscle activity changes might activate the muscle pump mechanism and thus increase venous return and venous emptying and decrease the swelling in the lower legs [103]. Indeed, combined active movements produce higher blood flow velocities and would therefore be the most effective in eliminating venous stasis and promoting venous return [62]. The kinetics of venous return are dependent on contraction frequency, with variations in the relaxation periods between muscle contractions [16,64,67,68] according to the type (*e*.*g*., walking, standing), intensity (*e*.*g*., speed), and duration of the exercise [64]. Although the level of agonist-antagonist muscle co-contraction is dependent on gait velocity [95], it appears that the lower muscle co-contraction in the lower leg with unstable shoes compared to barefoot during standing may be useful for the venous return mechanism and consequently would decrease lower leg circumferences (*i*.*e*., venous stasis).

It thus seems that the change in lower leg circumferences depends on the features of the shoe (barefoot, stable, unstable, athletic shoes), which induce modifications in the muscle activity in the form of dynamic contractions, and the characteristics of the exercise (*e*.*g*., floor interface, type, duration …). Indeed, Lin et al. [45] showed that floor type and standing duration also significantly affect lower leg (*e*.*g*., shank) circumferences.

### Quality

The heterogeneous data and good (n = 8) or fair (n = 5) methodological quality of the included studies nevertheless precluded definitive conclusions being drawn. Importantly, this systematic review highlights that further studies are needed to better identify and understand the effects of a range of shoe types on venous status. Future experiments in this area will need to be randomized and controlled and should feature the current shoe models or new shoe models that have not yet been studied. The focus should be on venous return for athletes and patients with chronic venous insufficiency or at risk of venous thrombosis, for example.

## Conclusion

Lower limb venous status is linked with variations in blood flow, venous pressures and lower limb circumferences, which can be modified by the shoe type. The features of unstable shoes (with rockers) or shoes with similar technology (heelless), athletic or soft shoes (air-cushion, air-sole), and customized foot orthotics can improve these venous variables. The features of shoes like high heels (stiletto, platform) and firm shoes and ankle joint immobilization can degrade these venous variables. The variations in venous status can be explained as follows. Shoe types are associated with several changes in the kinetic and kinematic variables and the muscle activity in the lower limbs; the efficiency of the lower limb skeletal muscle pump and foot pump are likely linked to gait kinetics and kinematics; the kinetics of venous return and the regional blood flow distribution are dependent on muscle contraction frequency and muscle fiber types, respectively; and exercise induces several venous changes that are dependent on the type, intensity and duration of the exercise, as well as the physical and physiological conditions of the subjects. It thus seems that it is important to consider the combination of shoe features, subject characteristics and the exercise conditions to interpret the findings about variations in venous variables. However, the data of these 13 studies showed great heterogeneity concerning the type of shoe, the duration or type of exercise and especially the venous parameters, which probably affected our ability to pool data and draw definitive conclusions from the studies within each category of venous variables. Consequently, this systematic review defined the types of shoes or the shoe features that might be better or less effective for venous return, but it was difficult to rank the shoes. Thus, given the heterogeneity in the data, the limited number of studies on each type of shoe, and the fair quality of some of the studies, further research is needed to reach greater consensus on how shoe types affect venous return in the lower limbs. Further studies could evaluate, for example, the effects of new types of shoes on venous return, like the so-called “recovery" shoes that are now used by many athletes after physical activity.

## Supporting information

S1 Checklist PRISMA checklist. (DOC)

S1 Fig Flow diagram of study selection. Figure presents the flow of information through the different phases (identification, screening, eligibility and inclusion) of a systematic review.
(DOC)
