# Supplementary material for: The effects of shoe type on lower limb venous status during gait or exercise: A systematic review
Source: PLoS One. 2020 Nov 25;15(11):e0239787. doi: 10.1371/journal.pone.0239787 (PMC7688113; doi:10.1371/journal.pone.0239787)
Supplement: S1 Fig — Figure presents the flow of information through the different phases (identification, screening, eligibility and inclusion) of a systematic review. (DOC) [file pone.0239787.s002.doc]

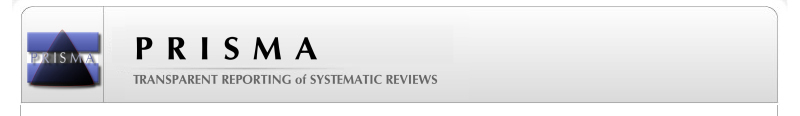
**PRISMA 2009 Flow Diagram**

**Figure 1.** Flow diagram of study selection.

**Screening**

**Included**

**Eligibility**

**Identification**

Records identified through database searching
(n =366)

Additional records identified through other sources
(n =5)

Records after duplicates removed
(n =311)

Records screened
(n =311)

Records excluded
(n =289)

Full-text articles assessed for eligibility
(n =22)

Full-text articles excluded,
(n =9) with reasons of validation of method (n =1), technique of running (n =1), microcirculation and mechanical pressure (n =1), micro-mobile foot compression (n =1), data unclear (n =1), same content (n =2), blood content, hematological data (inappropriate data) (n =2)

Studies included in qualitative synthesis
(n =13)

Studies included in quantitative synthesis (systematic review)
(n =13)

Figure presents the flow of information through the different phases (identification, screening, eligibility and inclusion) of a systematic review.
